# Investigation of the Behavior of Hardening Masonry Exposed to Variable Stresses

**DOI:** 10.3390/ma11050697

**Published:** 2018-04-28

**Authors:** Tomas Šlivinskas, Bronius Jonaitis, Jonas Gediminas Marčiukaitis, Robertas Zavalis

**Affiliations:** Department of Reinforced Concrete Structures and Geotechnics, Vilnius Gediminas Technical University, Saulėtekio al. 11, LT-10223 Vilnius, Lithuania; bronius.jonaitis@vgtu.lt (B.J.); jonas.gediminas.marciukaitis@vgtu.lt (J.G.M.); robertas.zavalis@vgtu.lt (R.Z.)

**Keywords:** masonry, deformations, masonry creep, variable long-term load, shrinkage

## Abstract

This paper analyzes the behavior of masonry under variable loads during execution (construction stage). It specifies the creep coefficient for calcium silicate brick masonry, presenting the research data of masonry deformation under variable and constant long-term loads. The interaction of separate layers of composite material in masonry is introduced and the formulae for determining long-term deformations are offered. The research results of masonry’s compressive strength and deformation properties under variable and constant long-term loads are presented. These are then compared to calculated ones. According to the presented comparison, the calculated long-term deformations coincide quite well with those determined experimentally.

## 1. Introduction

One of the oldest materials used in construction is masonry. Masonry is a material made of masonry units and mortar. According to its structure, masonry is made up of composite materials. The properties of composite materials depend on the mechanical properties of their separate components and interactions between the layers. Often, masonry is used in buildings with other materials, for example, with concrete. In such cases, the overall compatibility of structures is very important, i.e., compatibility of deformations, especially under long-term loads. Masonry stresses are often analyzed in transient design situations, i.e., during execution [1,2]. Unlike other materials, masonry structures are gradually loaded from the beginning. Self-weight loads of masonry and the weight of other constructions begins to act on the mortar of bed joints, reaching its designed strength [3,4]. When mortar hardens during the execution, masonry is affected by the variable long-term load. In the beginning of loading on the hardening masonry, the plastic deformations appear. After the design strength is reached, masonry is affected by the constant long-term load (persistent design situation). The research done by us and other authors [1,5,6,7,8,9] show that mechanical characteristics of masonry-mortar bed joints are different to those in benchmark mortar samples. According to [7,9], deformational properties of bed joints are 10–20 times higher than mortar deformation of benchmark mortar samples. At the contact area, the compressive strength of mortar and elasticity modulus are close to zero, but in the middle part of joint thickness, the characteristics of mortar get closer to the values of mechanical characteristics, which were estimated during the tests of benchmark mortar samples [2,10].

The creep is characteristic to masonry, as well as to concrete, i.e., the tendency to deform for a long time under the influence of a constant load. Creep is a very important feature in evaluating the distribution of stresses [11,12]. Other authors argue as well that masonry creep deformations heavily influence the distribution of stresses which affect masonry during a structure’s lifetime [13,14,15,16,17]. Creep redistribution of stresses in brickwork walls will result in reducing the stress on the vertical mortar joints and increasing it on horizontal bed joints [18,19]. As shown by [20,21], creep deformations may be several times bigger than those occurring from a short-time effect. Creep characteristics of materials depend on the mechanical properties of the material. In many cases, the properties of separate layers vary differently with time under loads. The change of properties of components determines the characteristics of creep on the whole composite unit. Such behavior is specific to masonry because of the age of masonry units and mortar. Therefore, the mechanical properties as well are different during the load-time.

The factors influencing creep deformations [22] which determine creep deformations of concrete type materials influence masonry as well. Such factors as masonry unit mechanical characteristics, masonry unit adsorption speed, mortar joint type, relative humidity, and mortar resistance influence masonry creep. The main factors which determine the development of creep deformations in masonry are the masonry-load age until masonry reaches the design strength and intensity of the long-term load, i.e., compressive-stress ratio with compressive strength [23,24].

The creep coefficient is one of the main parameters that is recommended by the normative documents [25] to count the deformation of structures under long-term loads. However, there is currently no methodology to estimate masonry creep in such documents. It should be noted that in this literature, only the range for some masonry units’ final creep coefficient are presented. The final creep coefficient for a ceramic unit’s masonry varies from 0.5 to 1.5 for calcium silicate, from 1.0 to 2.0 for lightweight aggregate concrete, from 1.0 to 3.0 etc. [25]. According to other standards [26], the values of creep coefficients vary from 1.5 to 3.0 respectively. The values of creep coefficients for clay bricks presented by [27] vary at the limits of 0.4–1.3 for the calcium silicate and 1.1–1.9. The presented range of creep coefficient values are not in any way related to the basic parameters which determine masonry creep deformations. In the same documents [25], it is recommended to estimate the creep coefficient during testing, but the creep tests are often complicated and take too long to apply in practice.

The research of [28] shows that the mechanical properties of loaded composite units stay constant over time when the constant strength is reached. However, the masonry is loaded gradually while the mortar has not reached the design strength because of the building technology features. Therefore, deformational properties of mortar change over time.

The creep of masonry is analyzed most often when masonry is affected by the long-term load after reaching the design strength (persistent design situation) [20,22]. However, there is a lack of information available on creep deformations of masonry as a layered composite under variable long-term loads during the construction stage, when the compressive strength of mortar is changing (in transient design situations). Therefore, the purpose of this paper is to analyze the change of the deformational properties of masonry as a composite material in transient design situations (during the execution of building construction) and under long-term constant loads when the masonry has reached its design strength (persistent design situation). The methodology to calculate deformations under variable and constant long-term loads by evaluating the effective modulus of elasticity of mortar bed joint is presented in this this paper.

## 2. Principles for Determining the Masonry Creep Parameters

Creep deformations depend on many factors: the value of the load and its nature of operation, intensity of load, mechanical and physical properties of materials, environmental impact, and others. On the basis of the studies carried out in [28], the following assumptions can be made when describing creep deformations of layered composites:

- linear dependence is in force between stresses and deformations of material being loaded;
- linear dependence is in force between creep deformations and the stresses which induce them;
- the principle of adding incremental actions may be applied to estimate creep deformations.

The creep deformations of materials depend on the loading moment and the nature of the load. If at the time $\tau =\tau_{1}$ the stresses are loaded $\sigma_{z}=\sigma_{z}\left(\tau_{1}\right),$ which vary over time, i.e., $\sigma_{z}\;\left(\tau_{1}\right)\neq const$, then the full relative deformations of layer i, $\varepsilon_{z,i}\left(t\right)$ can be described by well-known theoretical mechanics formula

(1) $$\varepsilon_{z,i}\left(t\right)=\sigma_{z}\left(\tau_{1}\right)\delta \left(t,\tau \right)+\int_{\tau_{1}}^{t}\frac{d\sigma_{z}}{d\left(t\right)}\;\delta \left(t,\tau \right)\;d\tau .$$

while integrating into parts, the formula is derived:

(2) $$\varepsilon_{z_{i}}\left(t\right)=\frac{\sigma_{z}\left(t\right)}{E_{i}\left(t\right)}-\int_{\tau_{1}}^{t}\sigma_{z}\left(\tau \right)\frac{\delta }{\delta_{\tau }}\delta \left(t,\tau \right)d\tau .$$

The first member of right side of the equation is elastic deformations which are caused by the compressive stresses $\sigma_{z}\left(t\right)$, which act in the time *t*. The second member evaluates the change of creep deformation and material properties over time in the layer *i*.

Creep deformations over time $\tau_{1}\leq \tau \leq \infty$:

(3) $$\frac{\partial \delta \left(t,\tau \right)}{\delta \tau }=\frac{\partial }{\delta \tau }\left[\frac{1}{E_{i}\left(\tau \right)}+C_{i}\left(t,\tau \right)\right],$$

where $C_{i}\left(t,\tau \right)=\frac{\varphi_{i}\left(t,\;\tau \right)}{E_{i}\left(\tau \right)}$—creep parameter of layer *i* material at the time *t*, $\varphi \left(t,\tau_{1}\right)$—the creep coefficient.

This case would correspond to the behavior of masonry layer *i* under variable loads and the properties of materials during action until the moment when the masonry reaches its design strength. After this, it is affected by the operating load, the stresses of which remain practically unchanged over time, i.e., $\sigma_{z}=const.$

At the time τ the masonry is loaded by the stresses which remain unchanged over time and the assumption is made that the properties of materials remain almost unchanged over time, i.e., $E_{t}\approx const$, the relative deformations of material of each layer under long-term load at the moment of time *t* are:

(4) $$\varepsilon_{z,i}\left(t,\tau_{1}\right)=\sigma \left(\tau_{1}\right)\delta \left(t,\tau_{1}\right)=\frac{\sigma \left(\tau_{1}\right)}{E_{i}\left(t,\tau_{1}\right)}=\frac{\sigma_{zi}\left(\tau_{1}\right)}{E_{i}(\tau_{1}/\left(1+\varphi_{i}(t,\tau_{1}\right)}\;=\sigma_{zi}\left(\tau_{1}\right)\left[\frac{1}{E_{i}\left(t_{0}\right)}+\frac{\varphi_{i}\left(t,\tau_{1}\right)}{E_{i}\left(\tau_{1}\right)}\right]=\sigma \left(\tau_{1}\right)I_{i}\left(t,\tau_{1}\right),$$

where $I_{i}\left(t,\tau_{1}\right)$ is the creep function of material layer *i*.

**Figure 1:**
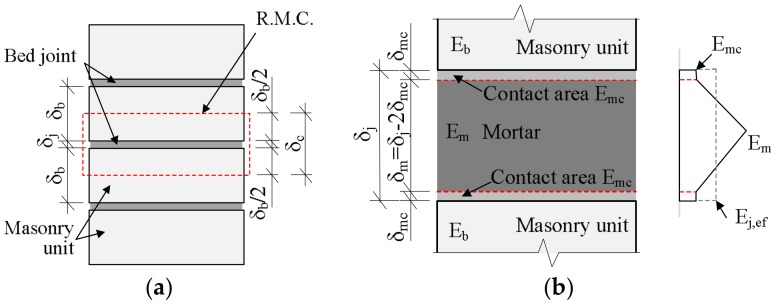
Masonry deformation scheme: (**a**) scheme of deformation of masonry fragment with bed joint affected by the longitudinal load fragment; (**b**) scheme of representative masonry cell (RMC), bed joint and distribution of mortar elasticity modulus in masonry bed joint.

Masonry deformations under the influence of compressive loads can be expressed using the scheme in Figure 1. As mentioned above, masonry is characterized by the fact that the zone of bed joints’ contact with masonry units has a heavy influence over the deformations. The overall deformations of masonry fragments being compressed (Figure 1) made of masonry units and bed joints (while evaluating the contact zone) would be equal to the sum of separate layers‘ deformations, i.e.,

(5) $$\Delta_{c}=\Delta_{b}+\Delta_{m}+2\Delta_{mc},$$

where $\Delta_{b},\;\Delta_{m}\;and\;\Delta_{mc}$—deformation of respectively masonry unit, masonry bed joint mortar, masonry unit and bed joint contact zone.

*E_m_* is the mortar elasticity modulus estimated during the tests of benchmark samples. *E_j,ef_* is the effective modulus of elasticity of the bed joint. It is estimated by the experiments using the dependence of bed joint stresses and deformations, when compressive stresses *σ_c_ = σ_u_*/*3*.

The deformations of masonry bed joint and contact zone can be expressed by total deformations $\Delta_{j}$, i.e.,:

(6) $$\Delta_{m}+2\Delta_{mc}=\Delta_{j.}$$

Since $\Delta_{i}=\varepsilon_{i}\delta_{i}$ (where $\varepsilon_{i}$ and $\delta_{i}$ are relative deformation of respective layer and thickness of layer), the equation can be written as follows:

(7) $$\varepsilon_{c}\delta_{c}=\varepsilon_{b}\delta_{b}+\varepsilon_{j}\delta_{j}.$$

When dividing both sides of the equation by total thickness of masonry representative masonry cell (RMC) $\delta_{c}$ we obtain the relative deformations of masonry:

(8) $$\varepsilon_{c}=\varepsilon_{b}\frac{\delta_{b}}{\delta_{c}}+\varepsilon_{j}\frac{\delta_{j}}{\delta_{c}}.$$

If we take the parts of separate layers as volumes ratios, i.e., $V_{b}=\frac{\delta_{b}l}{\delta_{c}l}$; $V_{m,ef}=\frac{\delta_{j}l}{\delta_{c}l}$, where *l* is the width of an element, the equation can be written as follows:

(9) $$\varepsilon_{c}=\varepsilon_{b}V_{b}+\varepsilon_{j}V_{j}.$$

If the masonry deformations are determined after a certain time *t*:

(10) $$\varepsilon_{c}=\varepsilon_{b}\left(t\right)V_{b}+\varepsilon_{j}\left(t\right)V_{j}.$$

A known expression of creep coefficient $\varphi \left(t,\tau_{1}\right)$ is:

(11) $$\varphi \left(t,\tau_{1}\right)=\frac{\varepsilon_{i}\left(t,\tau_{1}\right)}{\varepsilon_{0}}=\frac{\varepsilon_{i}\left(t,\tau_{1}\right)}{\sigma \left(\tau_{1}\right)}E\left(\tau_{1}\right)$$

In general, overall deformations of separate layers at a certain moment of time *t* are determined using the condition (4) by the following equation:

(12) $$\varepsilon_{i}\left(t,\tau_{1}\right)=\sigma \left(\tau_{1}\right)[\frac{1}{E_{i}\left(\tau_{1}\right)}+\frac{\varphi_{i}\left(t,\tau_{1}\right)}{E_{i}\left(t\right)}]=\sigma \left(\tau_{1}\right)I_{i}\left(t,\tau_{1}\right),$$

where $I_{i}\left(t,\tau_{1}\right)$ is a creep function.

Assuming that the longitudinal stresses in all layers are the same, that the modulus of elasticity of layers over time is constant, and that the bed joint modulus of elasticity is assumed as the effective modulus of elasticity $E_{i}\left(t\right)=E_{j,ef}\left(t\right)$ (Figure 1), the deformations of masonry $\varepsilon_{c}\left(t\right)$ and creep deformations $\varepsilon_{cr}\left(t\right)$ under long-term loads can be obtained from Equations (10) and (12):

(13) $$\varepsilon_{c}\left(t\right)=\sigma \left(\tau_{1}\right)\left[I_{b}\left(t,\tau_{1}\right)V_{b}+I_{j}\left(t,\tau_{1}\right)V_{j}\right].$$

(14) $$\varepsilon_{cr}\left(t\right)\;=\sigma \left(\tau \right)\left(\frac{\varphi_{b}\left(t\right)V_{b}}{E_{b}\left(t\right)}\;+\frac{\varphi_{j}\left(t\right)V_{j}}{E_{j,ef}\left(t\right)}\right).$$

In the case of variable mechanical properties and variable stresses of masonry, expression (14) can be written as follows:

(15) $$\varepsilon_{cr}\left(t,\tau_{1}\right)=\sigma \left(\xi \right)\left(\frac{\varphi_{b}\left(t,\tau_{1}\right)V_{b}}{E_{b}\left(t,\tau_{1}\right)}\;+\frac{\varphi_{j}\left(t,\tau \right)V_{j}}{E_{j,ef}\left(t,\tau \right)}\right),$$

where σ(ξ) are the equivalent stresses in the material of layer which cause the same deformations during the time t as the variable stresses at the same time.

Applying the obtained expressions (14) and (15) and using experimentally determined creep coefficients of masonry components, it is possible to estimate long-term deformations of masonry.

## 3. Experimental Research Program

**Figure 2:**
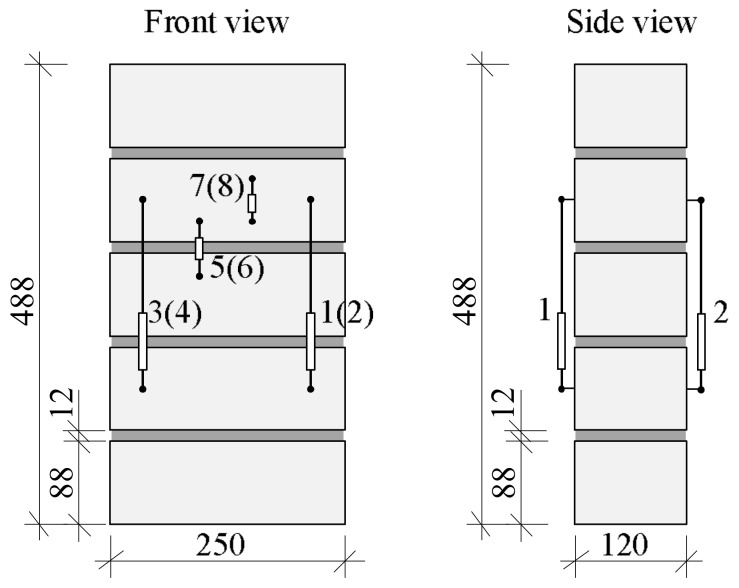
Scheme of deformation measurement points.

The behavior of calcium silicate brick masonry under long-term loads was analyzed by testing masonry samples made of five 250 × 125 × 88 mm masonry units and 11–12 mm thickness mortar without head joints. The average normalized compressive strength of masonry units is 22 N/mm^2^. The height of samples is 488 mm, the thickness of the bed joint is 12 mm (Figure 2). Longitudinal deformation of masonry was measured at points 1–4 (gauge length 200 mm), deformation of brick is measured at points 5 and 6 (gauge length 62 mm), and bed joints deformation at points 7 and 8 (gauge length 50 mm). The investigation of such samples allows us to test experimentally the behavior of bed joints (mortar and contact) under long-term loads.

General purpose mortar was used to lay bricks. The composition of Portland cement mortar was 1:4 (cement:sand) by the parts of volume. Quartz sand with granulometric composition till 2 mm was used. The ratio of water and cement *w*/*c* = 1.05 and mortar resilience according to cone penetration was 7 cm. The compressive strength of cement mortar is determined during the tests of benchmark samples according to the requirements of [29]. The mortar elasticity modulus is determined during the tests of prisms, the dimensions of which were 40 × 40 × 160 mm. Series M and series U are control samples of mortar and masonry units. B1–B8 series were made for the experimental research, with each of these consisting of three samples (Table 1).

**Figure 3:**
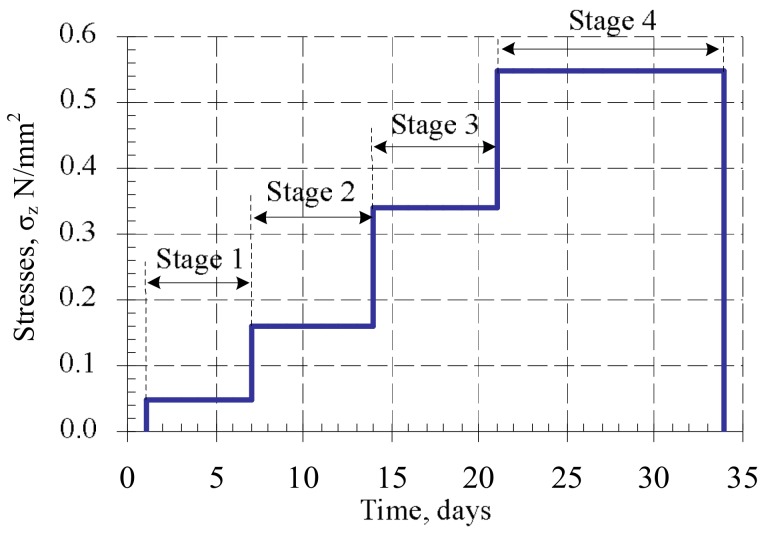
Long-term variable load loading schemes (series B7).

**Figure 4:**
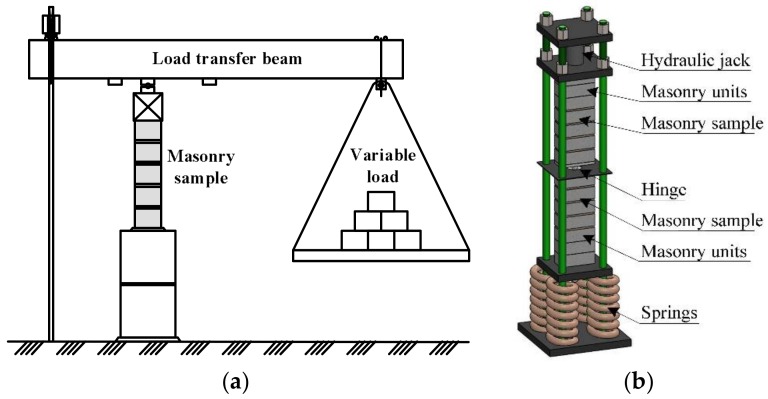
The scheme of loading: (**a**) The scheme of samples with long-term variable loads (series B7); (**b**) The scheme of samples with constant long-term loads (series B8).

Some of the samples (B7 series) were subjected to the variable load. The samples were loaded in stages, with periods ranging from 24 h to 34 days. The load intensity was close to the real building loads during execution (loads caused by the masonry’s own weight and variable loads). The scheme of application of compressive variable long-term loads in this research is presented in Figure 3. Variable loads were induced in the weighbridge (Figure 4). After 34 days, samples B7 were unloaded and tested under short-term static loads.

Variable loads were induced in the weighbridge (Figure 4). After 34 days, the load was eliminated from B7 samples and they were tested by short-term static loads.

Masonry samples (B8 series) were subjected to long-term constant loads after 34 days of curing (when masonry has reached its design strength). The duration of loading took 140 days (Figure 3b.) The intensity of the applied long-term load is expressed as $\eta =\sigma_{c}/f_{u}=0.3$, (where $f_{u}$ is compressive strength of masonry). The samples underwent the long-term load from a hydraulic jack (Figure 4).

Samples B7 and B8 were unloaded after 34 and 140 days, respectively, and they were tested by short-term static loads.

After testing the masonry samples from each series of vertical deformations of masonry, masonry units and bed joint were measured. In addition, the shrinkage strains of unloaded masonry and bed joint samples were observed. In order to estimate the change of masonry and mortar compressive strength and deformation properties over time, the control samples (B1–B6 series) were tested after 1, 2, 7, 14, 34, and 140 days.

Regarding compression strength of samples B5, the load intensity factor (*η*) for long-term loading (samples B8) was determined. Load intensity was equal to 30% of the failure load of samples B5.

## 4. Test Results and Analysis

**Figure 5:**
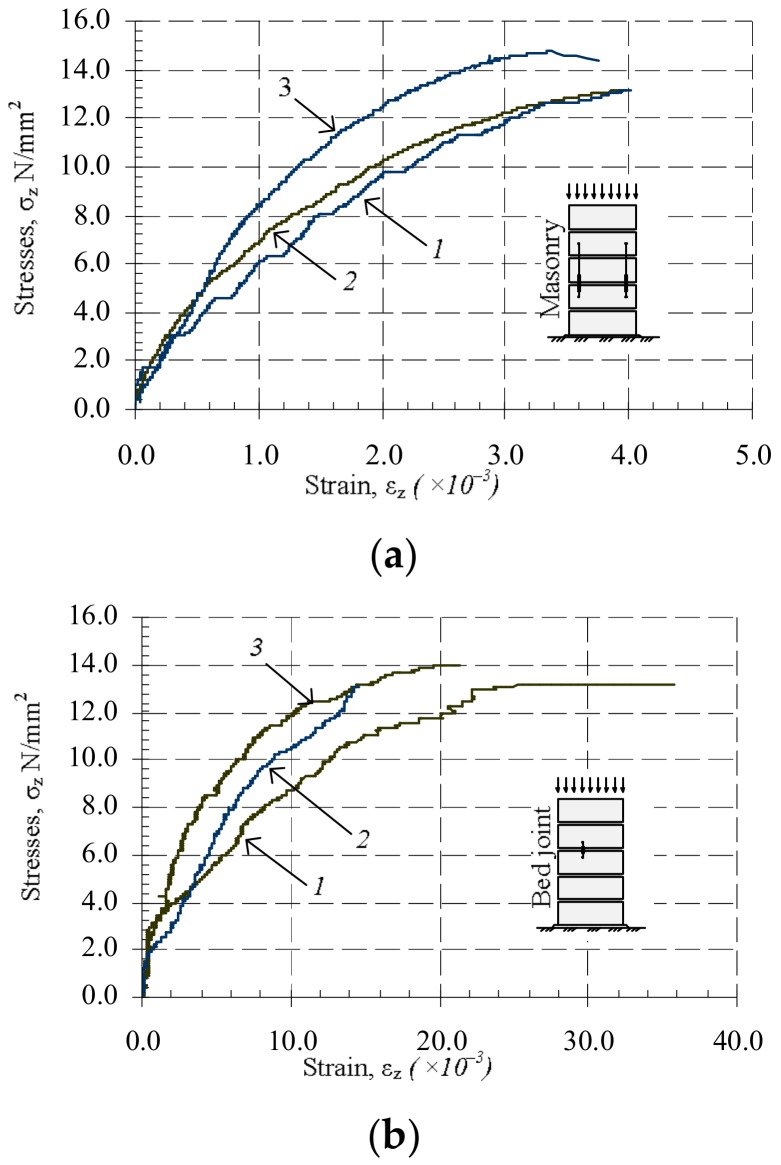
Relative average linear strains, 1—series B5; 2—series B6; 3—series B8: (**a**) masonry subjected to short-time load: 1—series B5; 2—series B6; 3—series B8; (**b**) masonry bed joint subjected to short-time load.

In order to determine the change of mortar and masonry mechanical properties (compressive strength $f_{u}$ and modulus of elasticity $E_{cm}$) over time, mortar and masonry samples were subjected to short-term static loads at different time. The results of the tests are presented in Table 2 and in Figure 5.

Our research showed that mortar compressive strength increased significantly at the initial stage of hardening. After seven days, mortar compressive strength was 7 N/mm^2^, and compared to mortar strength at two days age, increased 3.5 times. After 140 days of age, compressive strength practically did not change when compared with samples tested after seven days.

After seven days, mortar modulus of elasticity was 8 GPa, and compared to the modulus of elasticity of mortar after two days, increased 1.5 times. The change of the modulus of elasticity was insignificant after 14 and 34 days.

Masonry compressive strength after seven days of hardening increased up to 10% when compared to masonry compressive strength after two days. After 34 days, masonry compressive strength increased up to 10% when compared with masonry samples tested after seven days. The influence of variable long-term loads on masonry compressive strength is insignificant, with the compressive strength increasing up to 2% (comparing B7 and B5 series). The influence of constant long-term loads was higher. Masonry compressive strength increased up to 12% (comparing B8 and B6 series).

Masonry elasticity modulus after seven days of hardening, when compared to modulus after two days, increased to 13% (comparing B3 and B2 series). After 34 days, the masonry elasticity modulus increased up to 10% when compared with masonry samples tested after seven days (comparing B3 and B5 series). The influence of long-term variable loads for masonry elasticity modulus was insignificant (comparing B7 and B5 series). The influence of constant long-term loads was higher. Masonry compressive strength increased up to 40% (comparing B8 and B6 series)

Long-term load had more influence on the change of elasticity modulus. Joint-effective elasticity modulus, due to the influence of long-term constant loads (intensity *η* = 0.3 and period of 140 days), was doubled. This resulted in increased stiffness of mortar and masonry-unit contact zone under long-term loads. It can be argued that after contact zone deformations, the joint–mortar deformations determined the joint deformations. This statement is based on the residual bed joint deformations after the unloading of masonry samples. Most of the residual deformations were contact zone deformations.

**Figure 6:**
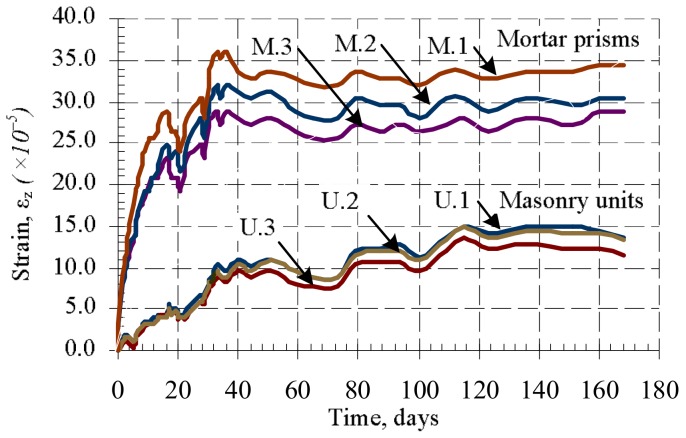
Relative average shrinkage–thermal strains of masonry units (separate samples of series U), and mortar (separate samples of series M).

**Figure 7:**
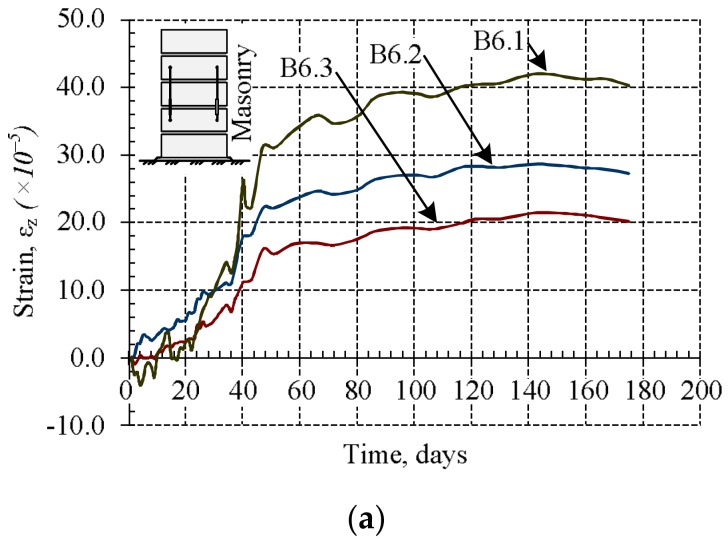

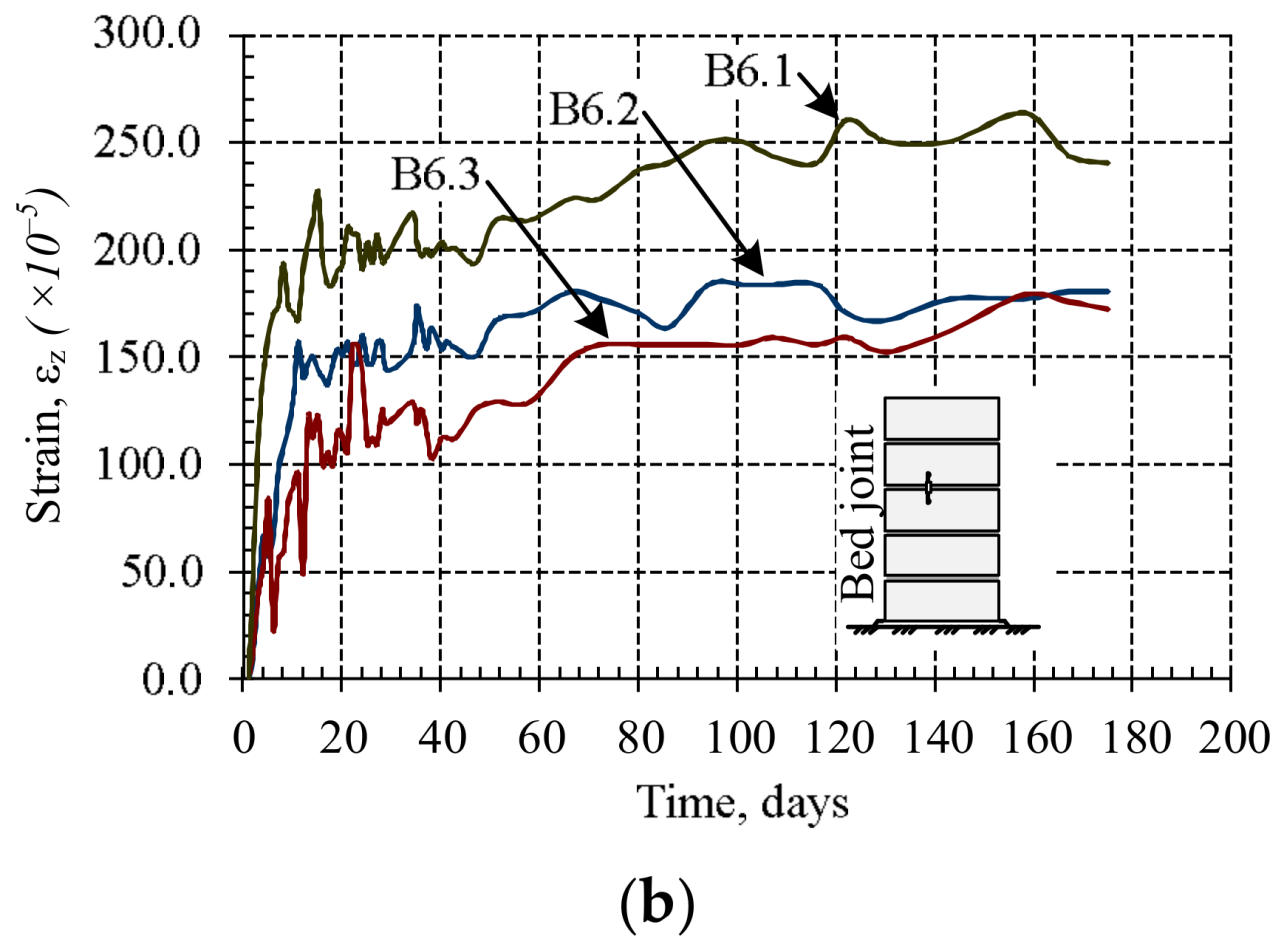
Relative average shrinkage–thermal strains (separate samples of B6 series): (**a**) of masonry; (**b**) of bed joints.

During the research masonry, masonry units‘, bed joints‘, and mortar shrinkage–thermal strains were measured for B6 samples. The measurements were started after 24 h passed from the formation of the samples. The results are presented in Figure 6 and Figure 7.

The results show that mortar shrinkage strains are significantly (2–2.5 times) bigger than masonry unit deformations (Figure 6). The shrinkage strains of masonry and bed joints were first measured as soon as the masonry samples were formed, with the results presented in Figure 7. At the first stage (up to 10 days), the masonry is expanding (Figure 7a), and at the same time, masonry bed joints shrink rapidly (Figure 7b). The expansion of masonry is related to the fact that masonry units take water from bed joint mortar rapidly. Later, some moisture from the masonry unit is released into the environment and comes back to the mortar, causing the masonry to shrinks [5].

The average shrinkage strains of bed joints are 5–6 times greater than the average shrinkage strains of masonry (Figure 7).

**Figure 8:**
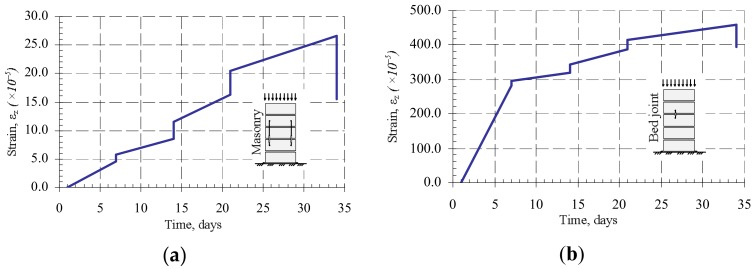
The dependence of the relative linear strains (Series B7): (**a**) masonry specimens compressed with the changing load over time; (**b**) bed joint of masonry specimens compressed with the changing load over time.

**Figure 9:**
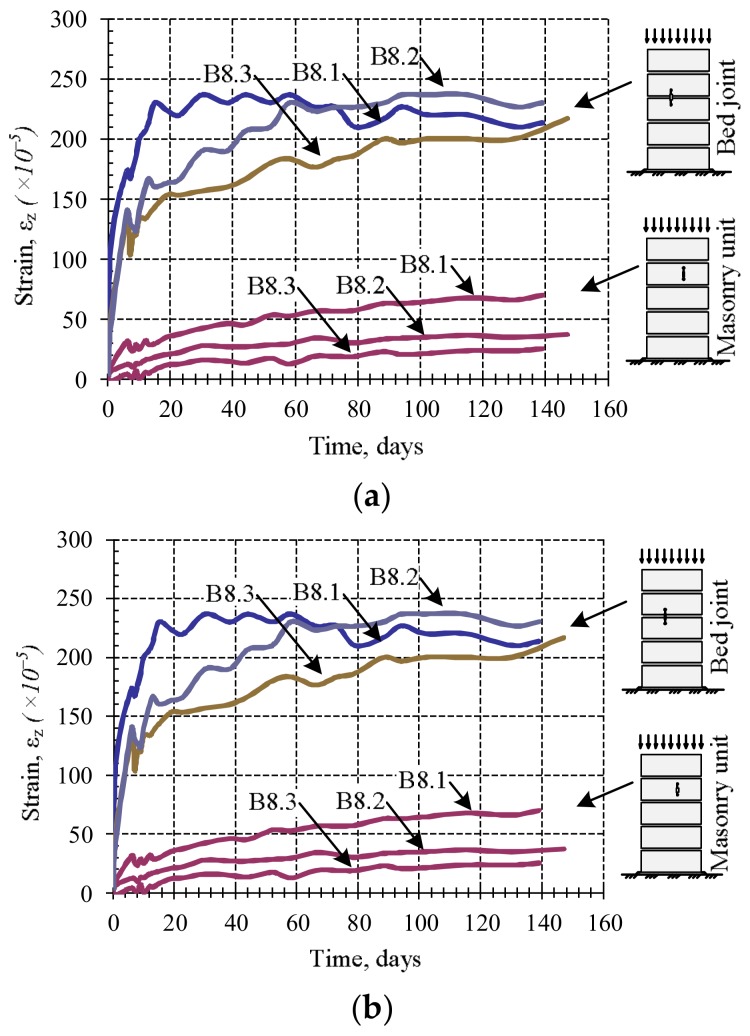
Relative creep deformations under long-term (separate samples of B8 series): (**a**) masonry bed joint and masonry unit; (**b**) masonry samples.

The results of the research of masonry fragments under loads of variable intensity are presented in Figure 8 and Figure 9. They show that if a constant load is maintained at the loading stages, then plastic (creep) deformations appear in masonry.

From Figure 8, it is possible to notice that the bed joint deformations form the greatest part of masonry plastic deformations. Due to the low intensity of the load during the studies of masonry subjected to variable long-term loads, plastic deformations of units did not appear. It is possible that the bed joint deformations determined the plastic masonry deformations (creep). This is associated with deformations of contact between mortar and masonry units.

From the diagrams (Figure 9), it can be seen that masonry creep deformations intensively develop at the first days of loading, corresponding to the first stage of creep, which in this case lasted about 10 days. The second stage of creep is observed during further investigation, and it lasts until the end of the study. The results show that the relative linear strains of masonry units are not significant compared to mortar joint deformations, therefore it can be concluded that the bed joint deformations determine the masonry longitudinal deformations.

The results of the study have shown that when mortar has reached its design strength, the deformations of masonry unit and bed joints determine the creep deformations of masonry. The creep coefficients of masonry units, masonry, and bed joints are presented in Table 3.

**Figure 10:**
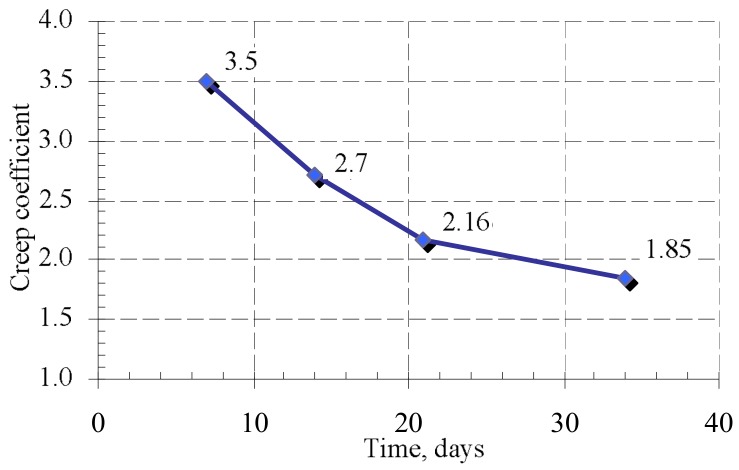
Change of creep coefficient under variable long-term loads.

Processing the data of the long-term study, the creep coefficient of masonry $\varphi_{mas}$, masonry unit $\varphi_{b}$, and bed joint $\varphi_{j}$ were estimated (Table 3). The masonry deformations under variable and constant long-term loads were estimated according to expressions (14) and (15). Creep coefficients $\varphi_{b}$ and $\varphi_{j}$ determined from experiments were used in current formulas. The obtained results are presented in Figure 10 and Table 4.

**Figure 11:**
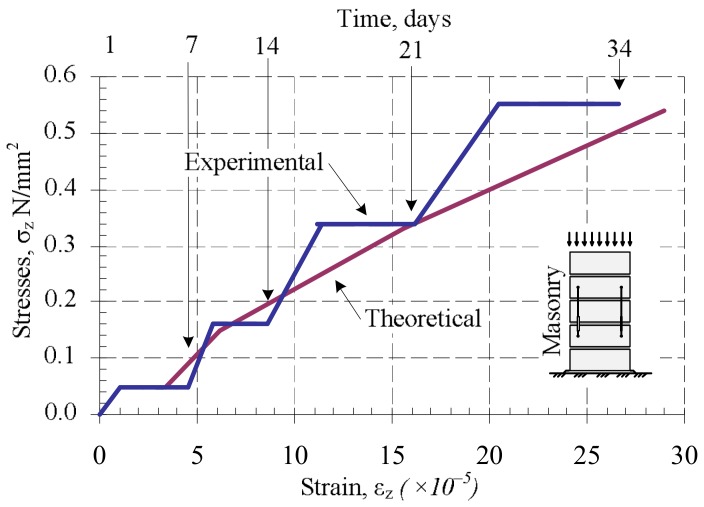
Dependence of plastic deformations on stresses in masonry under variable long-term loads: 1—experimental curve; 2—estimated curve.

After analyzing the creep deformations of masonry under variable loads, the change of the creep coefficient was determined when the intensity of long-term load and mortar strength increases. The results are presented in Figure 10. The studies have shown that the creep coefficient decreases over time. This can be explained by the fact that the compressive strength of mortar increases, and the mortar is thickened in the contact zone. The results show in Figure 11 that the creep (long-term) deformations of masonry under variable long-term loads calculated according to the Equation (15) coincide quite well with the deformations determined by the experiments. The calculation results were compared to the experimental ones, which are shown in Figure 11.

By applying Equation (15) to calculate composite creep deformations by estimating the experimental effective elasticity modulus of bed joints, obtained by experiment, the deformations of masonry which was compressed by long-term loads of variable intensity were determined.

## 5. Conclusions

Analysis of masonry behavior under long-term variable and constant loads was performed. Variable long-term loads were applied on fresh masonry (compressive strength of mortar is changing). Constant long-term loads were applied on masonry which has reached its design strength. Research and analysis of the results showed:

1. The influence of variable long-term loads (loading time $t-\tau_{1}=34$ days) on fresh masonry compressive strength is insignificant when compared to control samples. The influence of constant long-term loads (loading time $t-\tau_{1}=140$ days) on masonry which has reached the design strength before loading was higher. Compressive strength increased up to 12% when compared to control samples.
2. The influence of variable long-term loads (loading time $t-\tau_{1}=34$ days) on the fresh masonry modulus of elasticity is insignificant too regarding compressive strength. The influence of constant long-term loads (loading time $t-\tau_{1}=140$ days) on masonry which has reached its design strength before loading was higher. The modulus of elasticity increased up to 40% when compared to control samples.
3. The creep coefficient for masonry samples under variable long-term loads decreased from 3.4 to 1.95 during loading time ($t-\tau_{1}=34$ days). The change of variable load and change of mortar compressive strength influenced the creep coefficient. It is recommended to take the masonry creep coefficient of not less than three when analyzing masonry behavior in the construction stage (during execution).
4. The creep coefficient of masonry which has reached its design strength under the long-term loads (loading time $t-\tau_{1}=140$ days) is equal to two. This coincides with the upper limit of the creep coefficient recommended by Eurocode 6.
5. It is estimated that calculated creep deformations of masonry under loads of variable intensity and under constant long-term loads coincide quite well with the deformations which were determined by the experiments.

## Figures and Tables

**Table 1:** Sample series and the character of load.

Series	Age	Short-Term Test	Long-Term Test	Test Parameter
U	1 to 174	+	+	$f_{b}$; $E_{b}$; $\varepsilon_{sh}$
M	1 to 174	+	+	$f_{m}$; $E_{m}$; $\varepsilon_{sh}$
B1	1	+	–	$f_{mas}$; $E_{mas}$; $E_{j,ef}$
B2	2	+	–	$f_{mas}$; $E_{mas}$; $E_{j,ef}$
B3	7	+	–	$f_{mas}$; $E_{mas}$; $E_{j,ef}$
B4	14	+	–	$f_{mas}$; $E_{mas}$; $E_{j,ef}$
B5	34	+	–	$f_{mas}$; $E_{mas}$; $E_{j,ef}$
B6	174	+	–	$f_{mas}$; $E_{mas}$; $E_{j,ef}$; $\varepsilon_{sh}$
B7	1 to 34	+	+	$f_{mas}$; $E_{mas}$; $E_{j,ef}$; $\varepsilon_{cr}$
B8	34 to 174	+	+	$f_{mas}$; $E_{mas}$; $E_{j,ef}$; $\varepsilon_{cr}$

Notes: $f_{mas}$—masonry compressive strength; $E_{mas}$—masonry elasticity modulus; $E_{j,ef}—$masonry bed joint’ effective elasticity modulus; $\varepsilon_{cr}$—relative creep deformations; $\varepsilon_{sh}$—shrinkage thermal strains.

**Table 2:** The results of masonry units, mortar, and masonry mechanical properties test.

Series	Testing Age	Mortar Compressive Strength $f_{m}$, $N/mm^{2}$	Mortar Modulus of Elasticity *E_m_*. N/mm^2^	Masonry Compressive Strength $f_{mas}$, $N/mm^{2}$	Masonry Modulus of Elasticity $E_{mas}$, $N/mm^{2}$	Joint Effective Elasticity Modulus $E_{j,ef}$, $N/mm^{2}$
B1	1	—	—	11.08	3327	446
B2	2	1.95	5469	10.99	7516	786
B3	7	7.02	7993	12.13	8469	610
B4	14	6.04	7748	12.56	8244	1751
B5	34	6.78	8339	13.26	9307	899
B6	174	6.22	8890	12.46	8568	1200
B7	1–34 *	—	—	13.46	9164	1021
B8	34–174 **	7.22	9290	14.01	11,991	2400

Notes: * Sample series subjected to the variable long-term load, when age of sample $\tau_{1}=24$ h, and loading period $t-\tau_{1}=34$ days; ** Sample series subjected to long-term load, when age of sample $\tau_{1}=34$ days, and loading period $t-\tau_{1}=140$ days. Units; $N/{\mathrm{mm}}^{2}$.

**Table 3:** The experimentally determined creep coefficients for masonry units, bed joints, and masonry.

Series	Age	Masonry Creep Coefficient, $\varphi_{mas}$	Masonry Unit Creep Coef-Ficient, $\varphi_{b}$	Bed Joint Creep Coefficient, $\varphi_{m}$
B7	7	3.4	—	3.4
	14	2.7	—	2.7
	21	2.16	—	2.16
	34	1.85	—	1.85
B8	174	1.94	0.54	10.07

**Table 4:** Long-term deformations of masonry and elasticity modulus.

Series	Creep Coefficient	Relative Masonry Creep Deformations			
		Calculated (Equation (15))	Measured		
			B8.1	B8.2	B8.3
B8	1.94	7.72 × 10^−4^	7.67 × 10^−4^	6.53 × 10^−4^	8.11 × 10^−4^
	From 1.0 to 2.0 *	4.17 × 10^−4^ to 8.34 × 10^−4^ **			

* Creep coefficient according to the recommendations of Eurocode 6; ** Relative masonry creep deformations calculated using creep coefficient according to Eurocode 6.
